# The non-metabolizable glucose analog D-glucal inhibits aflatoxin biosynthesis and promotes kojic acid production in *Aspergillus flavus*

**DOI:** 10.1186/1471-2180-14-95

**Published:** 2014-04-17

**Authors:** Jin-Dan Zhang, Lida Han, Shijuan Yan, Chun-Ming Liu

**Affiliations:** 1 Key Laboratory of Plant Molecular Physiology, Institute of Botany, Chinese Academy of Sciences, Nanxincun 20, Beijing 100093, China

**Keywords:** D-glucal, D-galactal, Aflatoxin biosynthesis, *Aspergillus flavus*, Metabolomics

## Abstract

**Background:**

Aflatoxins (AFs) are potent carcinogenic compounds produced by several *Aspergillus* species, which pose serious threats to human health. As sugar is a preferred carbohydrate source for AF production, we examined the possibility of using sugar analogs to inhibit AF biosynthesis.

**Results:**

We showed that although D-glucal cannot be utilized by *A. flavus* as the sole carbohydrate source, it inhibited AF biosynthesis and promoted kojic acid production without affecting mycelial growth when applied to a glucose-containing medium. The inhibition occurred before the production of the first stable intermediate, norsolorinic acid, suggesting a complete inhibition of the AF biosynthetic pathway. Further studies showed that exogenous D-glucal in culture led to reduced accumulation of tricarboxylic acid (TCA) cycle intermediates and reduced glucose consumption, indicating that glycolysis is inhibited. Expression analyses revealed that D-glucal suppressed the expression of AF biosynthetic genes but promoted the expression of kojic acid biosynthetic genes.

**Conclusions:**

D-glucal as a non-metabolizable glucose analog inhibits the AF biosynthesis pathway by suppressing the expression of AF biosynthetic genes. The inhibition may occur either directly through interfering with glycolysis, or indirectly through reduced oxidative stresses from kojic acid biosynthesis.

## Background

Aflatoxins (AFs) are highly carcinogenic secondary metabolites produced by *Aspergillus* species such as *A. flavus* and *A. parasiticus* after invading plants or stored grains. Contaminations of these toxins in the food chain pose serious threats to humans and animals [1,2]. Previous studies focused on understanding the molecular machinery of AF biosynthesis [3], which have shown that most genes involved in the production of AF are located in a co-regulated gene cluster that encodes two regulatory proteins (*aflR* and *aflS*) and at least 26 down-stream metabolic enzymes [4]. An independently regulated sugar utilization gene cluster is located adjacently [5].

Some environmental factors and chemical reagents are known to be able to inhibit AF production [6,7]. Sugar is the most frequently used carbohydrate for studying AF production [8]. It has been proposed that the key factor determining if a carbohydrate supports AF production is its metabolic availability to the hexose monophosphate shunt and glycolysis pathway [9]. We thus speculate that sugar analogs that are unable to be utilized by *A. flavus* are candidate inhibitors for AF biosynthesis. Chemical analogs are often used to inhibit metabolism, as they may bind competitively to the active or allosteric sites of enzymes and hamper their activities [10,11]. Three glucose analogs, 2-deoxyglucose, α-methyglucoside and glucosamine, have been tested in *A. parasiticus* previously, but none of them inhibited AF production when applied to a glucose-containing medium [12].

D-glucal and D-galactal are cyclic enol ether derivatives of glucose and galactose, respectively (Additional file 1). In this study we examined in *A. flavus* for their effects on AF biosynthesis. It has been reported that D-glucal inhibits glucose oxidase (EC 1.1.3.4) [13-15], while D-galactal inhibits β-D-galactopyranosidase (EC 3.2.1.23) [16]. Whether these compounds have any effects on glycolysis and/or AF biosynthesis is not known. Results obtained in this study showed that D-glucal, but not D-galactal, is able to inhibit AF biosynthesis and to enhance kojic acid biosynthesis without affecting mycelial growth. The inhibition occurred before the production of norsolorinic acid (NOR), the first stable intermediate in the AF biosynthetic pathway. Metabolomics studies suggested that the glycolysis pathway was inhibited in mycelia grown in the presence of D-glucal. Using quantitative reverse transcription-PCR (qRT-PCR), we showed that exogenous D-glucal suppressed expression of AF biosynthetic genes tested but enhanced expression of kojic acid biosynthetic genes.

## Results

### Use of D-glucal and D-galactal as the sole carbohydrate source did not support mycelial growth

**Figure 1:**
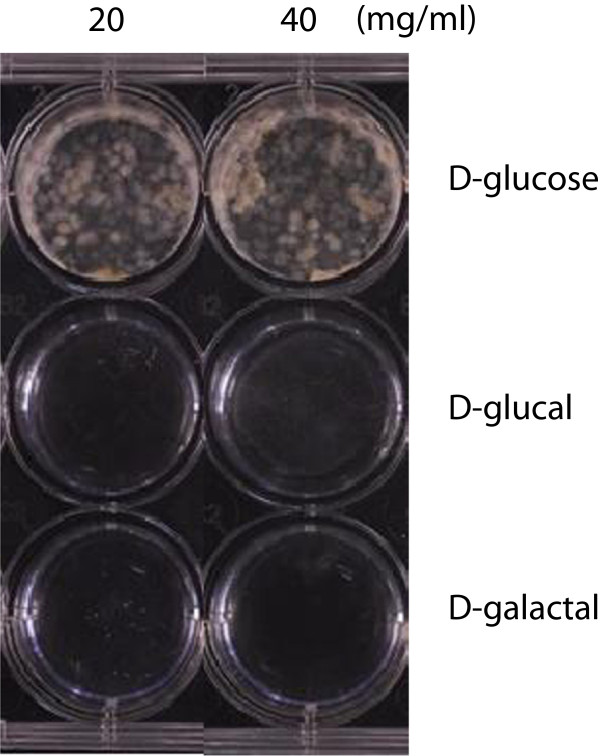
**D-glucal or D-galactal as the sole carbohydrate source did not support mycelial growth.***A. flavus* cultured for 3 d in GMS media in which glucose was replaced by 20 or 40 mg/mL D-glucal or D-galactal. GMS media containing 20 or 40 mg/mL D-glucose were used as controls. No visible mycelial growth was observed in D-glucal- or D-galactal-containing media.

The usual GMS medium used for culturing *A. flavus* contains 50 mg/mL glucose [17]. To examine if D-glucal and D-galactal could be used as the sole carbohydrate for mycelial growth, we replaced the glucose in the medium with 20 or 40 mg/mL D-glucal or D-galactal. Media containing either 20 or 40 mg/mL D-glucose were used as the control. After incubation of *A. flavus* A 3.2890 spores in these media for 3 d, we observed no mycelial growth in media with D-glucal or D-galactal, while abundant mycelial growth was observed in those two controls (Figure 1). No further growth was observed in media with D-glucal or D-galactal even when the incubation period was extended to 10 d, suggesting neither these two sugar analogs support mycelial growth when used as the sole carbohydrate.

### D-glucal inhibited AF biosynthesis and sporulation without affecting mycelial growth in GMS media

**Figure 2:**
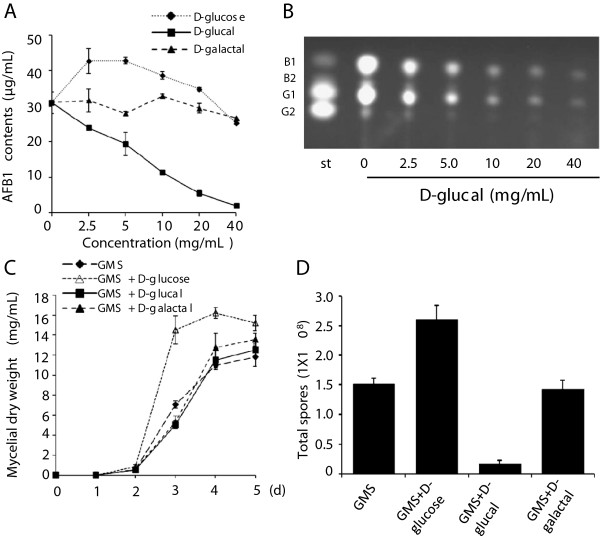
**Effects of D-glucal and D-galactal on AF production and sporulation. (A)** Amounts (μg per mL media) of AFB1 produced by *A. flavus* with different concentrations of D-glucose, D-glucal, or D-galactal (0, 2.5, 5, 10, 20 or 40 mg/mL). Data are presented as means ± S.D. (*n* = 3), from 3 independent experiments. **(B)** TLC analyses of AF production by *A. flavus* cultured in GMS media with different concentrations of D-glucal (0, 2.5, 5, 10, 20 or 40 mg/mL). **(C)** Growth curves of mycelia cultured in media with 40 mg/mL D-glucose, D-glucal, or D-galactal for 5 d. **(D)** Numbers of spores produced per mL culture with D-glucose, D-glucal, or D-galactal. Data are presented as means ± S.D. (*n* = 3).

To test whether D-glucal or D-galactal inhibit AF biosynthesis, spores of *A. flavus* A 3.2890 were inoculated in GMS liquid media (containing 50 mg/mL glucose) supplied with 2.5, 5, 10, 20, or 40 mg/mL of D-glucal or D-galactal and cultured at 28°C for 5 d. GMS media with the same amounts of additional D-glucose were used as controls. AFs were extracted from each sample, and the AFB1 contents were quantified using high pressure liquid chromatography (HPLC). As shown in Figure 2A, the AFB1 content was reduced significantly in samples with 2.5 to 40 mg/mL D-glucal. An almost complete inhibition was observed when 40 mg/mL D-glucal was used. In contrast, GMS media supplied with 2.5,5 or 10 mg/mL D-glucose promoted AFB1 production (Figure 2A). In samples supplied with D-galactal only a slight inhibition on AFB1 production was detected at the concentration of 40 mg/mL (Figure 2A). Using thin layer chromatography (TLC) analyses, we showed further that production of other AFs such as AFB1 and AFG1 were also inhibited by D-glucal (Figure 2B).

We next examined if D-glucal or D-galactal inhibited mycelial growth, and found that neither D-glucal nor D-galactal affected mycelial growth at the concentration of 40 mg/mL (Figure 2C). In contrast, additional D-glucose enhanced mycelial growth significantly, especially from the 3rd day onwards (Figure 2C). We next performed experiments on solid GMS media with 40 mg/mL D-glucal or D-galactal to assess if these sugar analogs have any effect on sporulation, and observed that exogenous D-glucal inhibited sporulation significantly*,* while additional D-glucose enhanced sporulation (Figure 2D). No effect was observed for D-galactal.

### D-glucal promoted kojic acid biosynthesis, but inhibited fatty acid biosynthesis and glucose consumption

**Figure 3:**
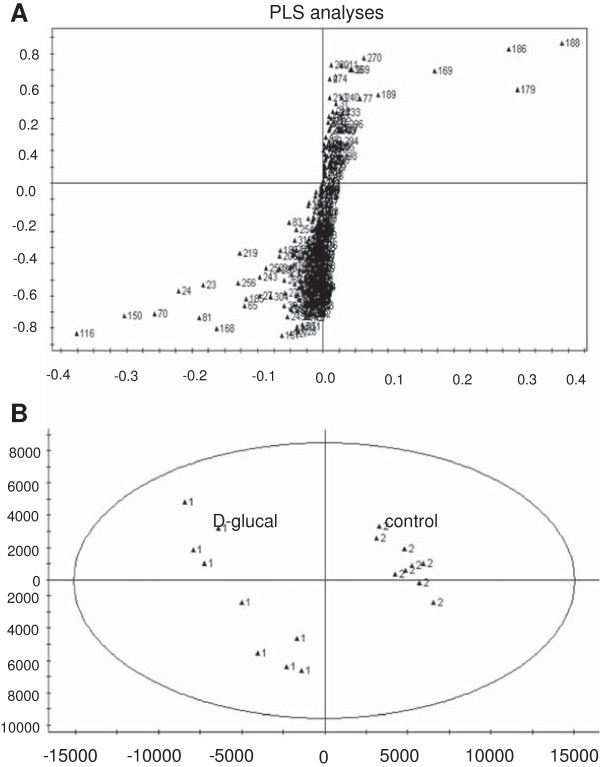
**Mycelia grown in media with or without D-glucal showed significant differences in the accumulation of various metabolites.** PLS analyses were performed using SIMCA-P V12.0. **(A)** Loadings plot obtained from PLS analyses of the entire GC-TOF MS dataset. **(B)** PLS scores plot of different metabolites in mycelia cultured for 5 d in GMS media with (▲1) or without (▲2) 40 mg/mL D-glucal, showing clear differences between these two sample groups. Nine replicates for each treatment.

We performed metabolomics analyses of mycelia of *A. flavus* A 3.2890 grown in media with or without 40 mg/mL D-glucal. The gas chromatography time-of-flight mass spectrometry (GC-TOF MS) based metabolomics technology developed in our lab has been shown to be a powerful tool to elucidate metabolic changes in *A. flavus*[18]. For statistical analyses, we used nine replicates for each treatment. Partial least-squares (PLS) analyses of metabolite peak areas showed clustering of two distinct groups for mycelia grown in media with or without D-glucal, suggesting that exogenous D-glucal imposed significant metabolic changes in mycelia (Figure 3). In particular, in the presence of D-glucal, the content of glucose, ribitol, glycerol and galactose were increased significantly, while the content of TCA intermediates (succinic acid, malic acid and fumaric acid) and fatty acids (FAs) including palmitic acid, stearic acid, oleic acid and linoleic acid were decreased (Table 1). We also noticed that, in the presence of D-glucal, the content of two secondary metabolites, kojic acid and furanacetic acid, were increased by 2 and 159 fold, respectively. These results together suggest that D-glucal interferes with both primary and secondary metabolism.

**Table 1:** **Metabolites with significant differences between mycelia of *A. flavus *** **grown in media with or without D-glucal (40 mg/mL)**

**Compounds**^**a**^	**Relative peak area**^**b**^		**Fold increase**^**c**^	***P *** **value**^**d**^
	**Control**	**D-glucal**		
**Organic acids**				
Furanacetic acid	0.0184 ± 0.0039	2.9291 ± 0.2771	159.10	<0.01
Kojic acid	0.0942 ± 0.0333	0.2076 ± 0.0293	2.20	<0.01
**Sugar metabolism**				
Ribitol	0.0066 ± 0.0038	0.0168 ± 0.0051	2.56	<0.01
Glycerol	0.0219 ± 0.0055	0.0514 ± 0.0350	2.34	<0.01
D-glucose	0.0133 ± 0.0060	0.1233 ± 0.0400	9.27	<0.01
D-galactose	0.0317 ± 0.0096	0.1750 ± 0.0743	5.53	<0.01
**TCA intermediates**				
Succinic acid	0.0053 ± 0.0016	0.0020 ± 0.0005	0.37	<0.01
Malic acid	0.0023 ± 0.0013	ND	ND	ND
Fumaric acid	0.0003 ± 0.0001	0.0002 ± 0.0000	0.53	<0.01
**Fatty acids**				
Palmitic acid	0.1428 ± 0.0116	0.0856 ± 0.0144	0.60	<0.01
Stearic acid	0.0702 ± 0.0150	0.0468 ± 0.0072	0.66	<0.01
Oleic acid	0.1957 ± 0.0159	0.0377 ± 0.0093	0.19	<0.01
Linoleic acid	0.2647 ± 0.0219	0.1281 ± 0.0212	0.48	<0.01
**Others**				
Glycine	0.0010 ± 0.0004	0.0004 ± 0.0002	0.39	<0.01
Pyrimidine	0.0018 ± 0.0005	0.0009 ± 0.0001	0.53	<0.01

^a^Individual metabolites were identified by GC-TOF MS, as described in the Methods. ^b^Relative peak area as normalized to the peak area of heptadecanoic acid. ^c^Folds represents the relative peak areas in D-glucal-treated samples/peak areas in the control. ^d^Statistical differences between control and D-glucal treated samples were calculated by two-tailed Student’s *t*-test. N.D.: not detected.

**Figure 4:**
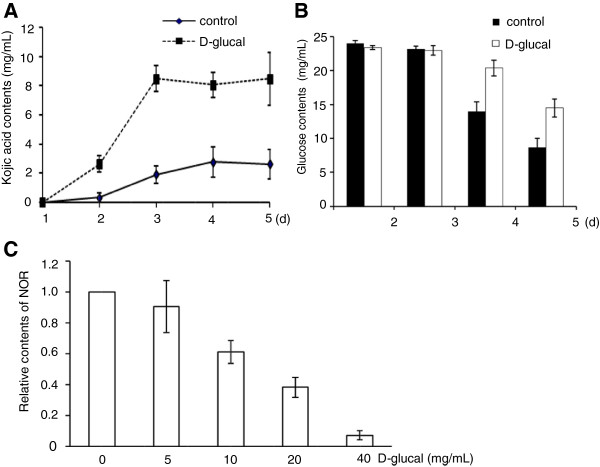
**Effects of D-glucal on kojic acid production, glucose consumption and NOR accumulation. (A)** Production of kojic acid by *A. flavus* grown in GMS media with or without 40 mg/mL D-glucal. Values are presented as means ± S.D. (*n* = 3), from three independent experiments. **(B)** Glucose contents in media at different time points when cultured in the presence of 40 mg/mL D-glucal. **(C)** D-glucal inhibited NOR accumulation. The amount of NOR in GMS medium lacking D-glucal was set to 1, those in other samples were calculated accordingly. Values are presented as means ± S.D. (*n* = 3).

We next cultured *A. flavus* A 3.2890 in GMS media with or without 40 mg/mL D-glucal, and measured kojic acid contents in media using a colorimetric method [19]. During the 5-d culture period the kojic acid contents in media with D-glucal were always higher (about 4 to 5 folds) than the control (Figure 4A). We also measured glucose content in the media and observed that, in the presence of D-glucal, the glucose content on the 4th and the 5th d were about 30% higher than those in the control media lacking D-glucal, suggesting that exogenous D-glucal inhibited the consumption of glucose (Figure 4B).

### D-glucal inhibited NOR production

We used the *A. flavus* strain Papa 827 to decipher at which step D-glucal inhibits AF biosynthesis. The lack of functional NOR reductase in this strain results in the accumulation of the first stable compound, NOR, in the AF biosynthetic pathway [20]. NOR is pinkish in color. After 4-d cultures, the control plate was pink in color, while no color was observed in the plate with 40 mg/mL D-glucal. Spectrophotometric analyses showed that NOR productions were significantly inhibited by D-glucal at concentrations of 10 mg/mL or higher (Figure 4C). These results suggest that D-glucal inhibits the AF biosynthesis pathway prior to the production of NOR.

### D-glucal inhibited expression of AF biosynthetic genes, but promoted expression of kojic acid biosynthetic genes

**Figure 5:**
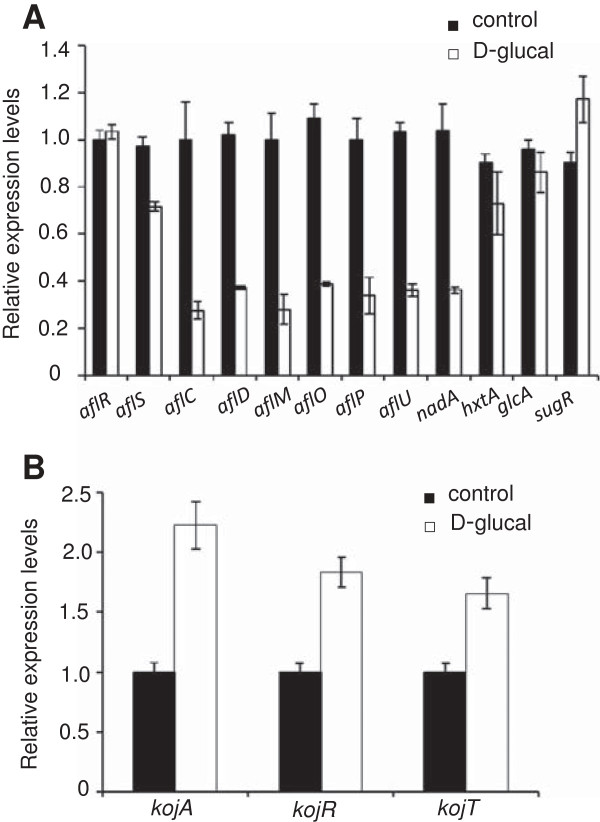
**Expression analyses of genes for AF and kojic acid production and sugar utilization. (A)** qRT-PCR analyses of expression of 9 AF biosynthetic genes (*aflR*, *aflS*, *aflC*, *aflD*, *aflM*, *aflP*, *aflO*, *aflU*, and *nadA*) and 3 sugar utilization genes (*hxtA*, *glcA* and *sugR*) in mycelia grown with or without 40 mg/mL D-glucal for 3 d, The relative expression levels were quantified through comparison with the expression level of *β-tubulin*. Data are presented as means ± S.D. (*n* = 3). **(B)** Expression of 3 kojic acid biosynthetic genes (*kojA*, *kojR*, *kojT*) by qRT-PCR in mycelia grown with or without 40 mg/mL D-glucal for 3 d. The relative expression levels were quantified through comparison with the expression level of *β-tubulin*. Data are presented as means ± S.D. (*n* = 3).

To examine the effect of D-glucal on AF biosynthesis at the transcriptional level, we analyzed expression of several genes in the AF biosynthetic gene cluster in *A. flavus* A 3.2890 by qRT-PCR and observed that, in the presence of 40 mg/mL D-glucal, no significant change was detected for *aflR* [a Zn (II)_2_ Cys_6_ transcription factor], while a 28% reduction was observed for *aflS* (a co-activator, Figure 5A). In addition, expression levels of all seven genes encoding AF biosynthetic enzymes tested, *aflC* (polyketide synthase), *aflD* (oxidoreductase), *aflM* (dehydrogenase), *aflO* (O-methyltransferase B), *aflP* (O-methyltransferase A), *aflU* (P450 monooxygenase) and *nadA* (a cytosolic enzyme converting AFB1 to AFG1), were decreased significantly (Figure 5A). Among these, *aflC* encodes an upstream enzyme in AF biosynthesis pathway, acting before NOR production to synthesize the polyketide backbone [21], while *nadA* encodes the most downstream enzyme, converting AFB1 to AFG1 [22,23].

We then examined if the expression levels of genes in the sugar utilization gene cluster were changed when cultured in media containing D-glucal. Of three genes tested, *sugR* (transcriptional regulator), *hxtA* (sugar transport), and *glcA* (glycosylation), none showed significant changes in expression (Figure 5A). We also analyzed the expression of genes involved in kojic acid biosynthesis: *kojR* [a Zn (II)_2_ Cys_6_ transcription factor], *kojA* (FDA-dependent oxidoreductase) and *kojT* (a major facilitator superfamily transporter) [24], and observed that expression levels of all these 3 genes were increased when cultured in media with 40 mg/mL D-glucal (Figure 5B).

## Discussion

Sugars such as glucose and sucrose are preferred carbohydrates for growth and AF production [25]. Glucose is utilized through glycolysis and TCA cycling to provide energy and substrates for downstream metabolic pathways including the AF biosynthesis pathway [26,27]. Glucose may also act as a signal molecule in sugar sensing to fine-tune the growth and metabolic activities based on the availability of glucose [28]. Genomic sequencing of *A. flavus* revealed 55 putative secondary metabolism gene clusters that are differentially regulated through global transcriptional regulators such as LaeA and VeA [2]. Individual secondary metabolic pathways may further be regulated independently by transcriptional regulators located in individual gene clusters for example, *aflR* and *aflS* in AF biosynthesis and *kojR* in kojic acid biosynthesis [2,29,30].

**Figure 6:**
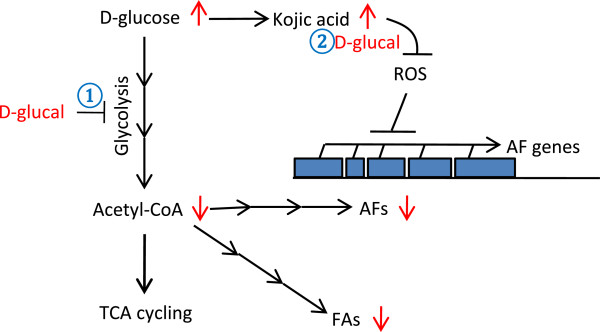
**A working model of D-glucal in inhibiting AF production.** A hypothetical model showing possible roles of D-glucal in inhibiting AF production. Routes ① and ② depict two possible modes of actions. For further explanations, see the Discussion.

Non-metabolizable chemical analogs have been used in the past to inhibit metabolic pathways and to study metabolism [25]. In this study, we examined D-galactal and D-glucal, non-metabolizable chemical analogs of D-glucose and galactose, respectively, for their effects on AF biosynthesis in *A. flavus*. We observed that 40 mg/mL D-galactal as a galactose analog did not have much effect on AF production. This is not surprising as though galactose supports mycelial growth, it cannot be utilized efficiently for AF biosynthesis [8,31], suggesting galactose utilization might be independent from the AF biosynthesis pathway. In contrast, 40 mg/mL D-glucal effectively inhibited AF biosynthesis. In the presence of D-glucal, glucose consumption and FA biosynthesis were reduced; the concentrations of TCA cycle intermediates were also reduced. In contrast, the production of kojic acid, a secondary metabolite produced directly from glucose, and furanacetic acid, a secondary metabolite of unknown function, were increased. At the metabolic level, we observed that D-glucal inhibited AF biosynthesis before production of the first stable intermediate, NOR. Based on these observations, we propose that, as depicted in route ① of Figure 6, D-glucal may interfere directly with enzymes such as hexokinase in glycolysis to prevent sufficient acetyl-CoA to be produced for TCA cycling, and for AF and FA biosynthesis in *A. flavus*. Consequently this has led to the increased glucose level observed in media and possibly in mycelia as well, which may enhance kojic acid biosynthesis. This hypothesis is in agreement with some previous observations that showed that active AF production usually correlates with increased accumulation of TCA cycle intermediates and active FA biosynthesis [26,32,33].

Alternatively, since kojic acid is synthesized from glucose without going through glycolysis [34], exogenous D-glucal may interfere with the sugar sensing machinery to enhance kojic acid production directly. The accumulation of kojic acid may have then relieved the oxidative stress in the fungus, which consequently inhibits AF biosynthesis at the transcriptional level, as depicted in route ② of Figure 6. It is known that kojic acid is a potent antioxidant that is able to scavenge reactive oxygen species [35], and oxidative stress is a prerequisite for AF production [36]. As reported previously, antioxidants such as eugenol, saffron and caffeic acid are able to inhibit AF biosynthesis [37-39]. A negative correlation between kojic acid and AF production has been shown before. D-xylose, ethanol, Dioctatin A and high temperature are factors known to promote kojic acid production, but inhibit AF biosynthesis [40,41].

We also showed that, although neither D-glucal nor D-galactal supported mycelial growth when used as the sole carbohydrate source, D-glucal inhibited sporulation without affecting mycelial growth. Secondary metabolism is usually associated with sporulation in fungi [42], a G-protein signaling pathway is involved in coupling these two processes [43,44]. The coupling does not seem to be very tight, as molasses promotes sporulation but suppresses AF production in *Aspergillus flavus*[45]. It will be interesting to study if D-glucal acts independently in AF production and sporulation, or if a common signaling pathway is involved in both processes.

## Conclusions

We showed in this study that D-glucal effectively inhibited AF biosynthesis and promoted kojic acid biosynthesis through modulating expression of genes in these two secondary metabolic pathways. The inhibition may occur either directly through interfering with glycolysis, or indirectly through reduced oxidative stresses from kojic acid biosynthesis.

## Methods

### Fungal strains and culture conditions

*A. flavus* A3.2890 was obtained from the China General Microbiological Culture Collection Center, Institute of Microbiology, Chinese Academy of Sciences. *A. flavus* Papa 827 was provided by Gary Payne [20]. All strains were maintained in glycerol stocks and grown on potato dextrose agar (PDA) medium at 37°C for 4 d before spores were collected to initiate new cultures. The PDA medium was also used for the examination of NOR accumulation. For all other experiments, Adye and Mateles’ GMS medium was used (containing 5% glucose) [17]. D-glucal and D-galactal were purchased from Chemsynlab (Beijing, China). AF standards were purchased from Sigma (St. Louis, USA).

### Determination of fungal dry weights

Mycelia cultured for 2, 3, 4 and 5 days were harvested by filtration through two layers of filter paper, washed by sterilized water, and freeze-dried before weighing.

### AF extractions and analyses

Mycelia grown in 1 mL GMS media were extracted using 1 mL chloroform/water (1:1). After vortexing for 2 min, the mixture was centrifuged at 12,000 rpm for 10 min. The organic phase was then collected and filtered through a 0.22 μm filter, dried under nitrogen gas, and re-dissolved in 200 μL chloroform before being analyzed by TLC as described previously [18]. The AFB1 content was measured by HPLC (Agilent 1200, Waldbronn, Germany) using a reverse phase C18 column (150 mm in length and 4.6 mm in internal diameter, 5 μm particle size, Agilent), eluted initially with 25% methanol/20% acetonitrile water solution for 3 min, and then with 38% methanol for 2.9 min, detected by a DAD analyzer at 360 nm. Quantifications were performed by measuring peak areas and comparing with an AFB1 standard calibration curve.

### Spore counting

Three mL of sterile water with 0.05% Tween-20 was added to the surface of PDA plates on which *A. flavus* were grown for 3 d. Spores were scraped with a cell scraper before being counted with a haemacytometer.

### qRT-PCR

Mycelia grown in GMS media with or without 40 mg/mL D-glucal for 3 d were collected and ground in liquid nitrogen, and total RNA was extracted using a Trizol solution (Invitrogen, CA, USA). PolyA mRNA was purified from mycelia with the PolyAT Rack mRNA isolation system (Promega, Madison, WI). Template cDNA was synthesized by reverse transcription with ReverTra Ace-α-® (Toyobo, Japan) at 42°C for 1 h, followed by incubation at 85°C for 15 min to terminate the reaction. qRT-PCR was performed using SYBR Green I (Takara, Japan) and a Rotor-Gene 3000 (Corbett, Australia) with primers described in Additional file 2: Table S1. PCR programs used are 94°C for 30 sec, 40 cycles at 94°C for 30 sec, followed by annealing (55°C for *aflO*, *aflR*, *aflS*, *aflD* and *β-tubulin*; 62.5°C for *aflU* and *nadA*; 58°C for *kojA*, *kojR* and *kojT*; 61°C for *hxtA*, *glcA* and *sugR*; 60°C for *aflC*, *aflM* and *aflP*) for 30 sec, and 72°C for 30 sec. The relative expression levels were quantified by comparing the expression level of *β-tubulin*.

### Kojic acid and glucose measurements

*A. flavus* A3.2890 was cultured in a GMS liquid medium plus 40 mg/mL D-glucal for 5 d. Media samples were harvested by centrifugation at 12,000 rpm for 10 min before kojic acid was quantified according to Bentley [19]. Glucose contents in media were measured by using a glucose determination kit (Applygen, Beijing). The absorbance was measured at 550 nm using a multimode plate reader (Tecan Infinite M200 PRO, Switzerland), and calculated against a glucose standard curve.

### Metabolomics analyses

Metabolites in mycelia of *A. flavus* A3.2890 cultured in a GMS liquid medium with or without 40 mg/mL D-glucal for 5 d were purified, silyl-derivatized and analyzed with GC-TOF MS as described previously [18], with minor modifications. The column temperature was held at 100°C for 3 min, and raised to 150°C at a rate of 10°C/min, then to 250°C at 5°C/min, finally to 300°C at 10°C/min, and held for 15 min at 300°C. PLS analysis was performed using SIMCA-P V12.0 (Umetrics, Sweden).

### NOR analyses

*A. flavus* Papa 827 was cultured for 4 d on PDA media containing 0, 5, 10, 20, or 40 mg/mL D-glucal. Quantification of NOR was performed as reported [46] with modifications. Briefly, media samples were mixed with 0.5 mL 90:10 methanol/1 N NaOH (pH 10). NOR is pinkish at this pH, which allows for spectrophotometric measurement at 595 nm with a 96-well Tecan plate reader.

### Statistical analyses

All experiments were conducted with at least 3 replicates and statistical significance was evaluated using Student’s *t*-tests.

## Competing interests

The authors declare that they have no competing interests.

## Authors’ contributions

JDZ designed and performed the experiments; JDZ and LDH analyzed the data; SJY helped to develop some analysis tools; JDZ and CML wrote the manuscript. All authors read and approved the final manuscript.

## Supplementary Material

Additional file 1 Structures of D-glucose, D-glucal and D-galactal. Click here for file

Additional file 2: Table S1 Primers used for qRT-PCR. Click here for file

## References

[B1] Yu J Cleveland TE Nierman WC Bennett JW *Aspergillus flavus* genomics: gateway to human and animal health, food safety, and crop resistance to diseases Rev Iberoam Micol 2005 22 4 194 202 10.1016/S1130-1406(05)70043-7 16499411

[B2] Amaike S Keller NP *Aspergillus flavus* Annu Rev Phytopathol 2011 49 107 133 10.1146/annurev-phyto-072910-095221 21513456

[B3] Roze LV Hong SY Linz JE Aflatoxin biosynthesis: current frontiers Annu Rev Food Sci Technol 2013 4 293 311 10.1146/annurev-food-083012-123702 23244396

[B4] Cleveland TE Yu J Fedorova N Bhatnagar D Payne GA Nierman WC Bennett JW Potential of *Aspergillus flavus* genomics for applications in biotechnology Trends Biotechnol 2009 27 3 151 157 10.1016/j.tibtech.2008.11.008 19195728

[B5] Yu J Chang P Bhatnagar D Cleveland TE Cloning of a sugar utilization gene cluster in *Aspergillus parasiticus* Biochim Biophys Acta 2000 1493 1–2 211 214 10978525 10.1016/s0167-4781(00)00148-2

[B6] Holmes RA Boston RS Payne GA Diverse inhibitors of aflatoxin biosynthesis Appl Microbiol Biotechnol 2008 78 4 559 572 10.1007/s00253-008-1362-0 18246345

[B7] Gupta SR Prasanna HR Viswanathan L Venkitasubramanian TA Effect of some inhibitors on aflatoxin-production in a synthetic medium and on the incorporation of acetate-1–^14^C into aflatoxins by resting mycelia of *Aspergillus parasiticus* Bull Environ Contam Toxicol 1976 15 4 447 453 10.1007/BF01685070 1260151

[B8] Davis ND Diener UL Agnihotr VP Production of aflatoxins B1 and G1 in chemically defined medium Mycopathol Mycol Appl 1967 31 3–4 251 256 6031300 10.1007/BF02053422

[B9] Davis ND Diener UL Growth and aflatoxin production by *Aspergillus parasiticus* from various carbon sources Appl Microbiol 1968 16 1 158 159 5636458 10.1128/am.16.1.158-159.1968 PMC547343

[B10] Gloster TM Zandberg WF Heinonen JE Shen DL Deng L Vocadlo DJ Hijacking a biosynthetic pathway yields a glycosyltransferase inhibitor within cells Nat Chem Biol 2011 7 3 174 181 10.1038/nchembio.520 21258330 PMC3202988

[B11] Araujo WL Trofimova L Mkrtchyan G Steinhauser D Krall L Graf A Fernie AR Bunik VI On the role of the mitochondrial 2-oxoglutarate dehydrogenase complex in amino acid metabolism Amino Acids 2013 44 2 683 700 10.1007/s00726-012-1392-x 22983303

[B12] Buchanan RL Ocker LA Stahl HG Effect of 2-deoxyglucose, alpha-methylglucoside, and glucosamine on aflatoxin production by *Aspergillus parasiticus* Arch Microbiol 1985 142 2 200 203 10.1007/BF00447068 4037981

[B13] Chenault HK Mandes RF Selective inhibition of metabolic enzymes by enzymatically synthesized D-glucal-6-phosphate Bioorg Med Chem 1994 2 7 627 629 10.1016/0968-0896(94)85010-0 7858968

[B14] Rogers MJ Brandt KG Multiple inhibition analysis of *Aspergillus niger* glucose oxidase by D-glucal and halide ions Biochemistry 1971 10 25 4636 4641 10.1021/bi00801a007 5140182

[B15] Rogers MJ Brandt KG Interaction of D-glucal with *Aspergillus niger* glucose oxidase Biochemistry 1971 10 25 4624 4630 10.1021/bi00801a005 5140180

[B16] Lee YC Inhibition of beta-D-galactosidases by D-galactal Biochem Biophys Res Commun 1969 35 1 161 167 10.1016/0006-291X(69)90499-9 4305272

[B17] Adye J Mateles RI Incorporation of labelled compounds into aflatoxins Biochim Biophys Acta 1964 86 2 418 420 10.1016/0304-4165(64)90077-7 14171025

[B18] Yan SJ Liang YT Zhang JD Liu CM *Aspergillus flavus* grown in peptone as the carbon source exhibits spore density- and peptone concentration-dependent aflatoxin biosynthesis BMC Microbiol 2012 12 106 10.1186/1471-2180-12-106 22694821 PMC3412747

[B19] Bentley R Preparation and analysis of kojic acid Method Enzymol 1957 3 238 241

[B20] Papa KE Genetics of *Aspergillus flavus*: linkage of aflatoxin mutants Can J Microbiol 1984 30 1 68 73 10.1139/m84-012 6424919

[B21] Feng GH Leonard TJ Characterization of the polyketide synthase gene (*pksL1*) required for aflatoxin biosynthesis in *Aspergillus parasiticus* J Bacteriol 1995 177 21 6246 6254 7592391 10.1128/jb.177.21.6246-6254.1995 PMC177466

[B22] Ehrlich KC Scharfenstein LL Montalbano BG Chang PK Are the genes *nadA* and *norB* involved in formation of aflatoxin G1? Int J Mol Sci 2008 9 9 1717 1729 10.3390/ijms9091717 19325828 PMC2635760

[B23] Cai J Zeng H Shima Y Hatabayashi H Nakagawa H Ito Y Adachi Y Nakajima H Yabe K Involvement of the *nadA* gene in formation of G-group aflatoxins in *Aspergillus parasiticus* Fungal Genet Biol 2008 45 7 1081 1093 10.1016/j.fgb.2008.03.003 18486503

[B24] Terabayashi Y Sano M Yamane N Marui J Tamano K Sagara J Dohmoto M Oda K Ohshima E Tachibana K Higa Y Ohashi S Koike H Machida M Identification and characterization of genes responsible for biosynthesis of kojic acid, an industrially important compound from *Aspergillus oryzae* Fungal Genet Biol 2010 47 12 953 961 10.1016/j.fgb.2010.08.014 20849972

[B25] Buchanan RL Stahl HG Ability of various carbon-sources to induce and support aflatoxin synthesis by *Aspergillus parasiticus* J Food Safety 1984 6 271 279 10.1111/j.1745-4565.1984.tb00488.x

[B26] Tyagi JS Venkitasubramanian TA The role of glycolysis in aflatoxin biosynthesis Can J Microbiol 1981 27 12 1276 1282 10.1139/m81-196 6460551

[B27] Shantha T Murthy VS Influence of tricarboxylic acid cycle intermediates and related metabolites on the biosynthesis of aflatoxin by resting cells of *Aspergillus flavus* Appl Environ Microbiol 1981 42 5 758 761 6797348 10.1128/aem.42.5.758-761.1981 PMC244103

[B28] Rolland F Winderickx J Thevelein JM Glucose-sensing and -signalling mechanisms in yeast Fems Yeast Res 2002 2 2 183 201 10.1111/j.1567-1364.2002.tb00084.x 12702307

[B29] Marui J Yamane N Ohashi-Kunihiro S Ando T Terabayashi Y Sano M Ohashi S Ohshima E Tachibana K Higa Y Nishimura M Koike H Machida M Kojic acid biosynthesis in *Aspergillus oryzae* is regulated by a Zn(II)(2)Cys(6) transcriptional activator and induced by kojic acid at the transcriptional level J Biosci Bioeng 2011 112 1 40 43 10.1016/j.jbiosc.2011.03.010 21514215

[B30] Yu JJ Fedorova ND Montalbano BG Bhatnagar D Cleveland TE Bennett JW Nierman WC Tight control of mycotoxin biosynthesis gene expression in *Aspergillus flavus* by temperature as revealed by RNA-Seq Fems Microbiol Lett 2011 322 2 145 149 10.1111/j.1574-6968.2011.02345.x 21707733

[B31] Pegg AE Poulin R Coward JK Use of aminopropyltransferase inhibitors and of non-metabolizable analogs to study polyamine regulation and function Int J Biochem Cell Biol 1995 27 5 425 442 10.1016/1357-2725(95)00007-C 7641073

[B32] Buchanan RL Federowicz D Stahl HG Activities of tricarboxylic-acid cycle enzymes in an aflatoxigenic strain of *Aspergillus parasiticus* after a peptone to glucose carbon source shift T Brit Mycol Soc 1985 84 Mar 267 275

[B33] Maggon KK Gupta SK Venkitasubramanian TA Biosynthesis of aflatoxins Bacteriol Rev 1977 41 4 822 855 23090 10.1128/br.41.4.822-855.1977 PMC414029

[B34] Arnstein HR Bentley R The biosynthesis of kojic acid. I. Production from (1-^14^C) and (3:4-^14^C2) glucose and (2-^14^C)-1:3-dihydroxyacetone Biochem J 1953 54 3 493 508 13058934 10.1042/bj0540493 PMC1269023

[B35] Gomes AJ Lunardi CN Gonzalez S Tedesco AC The antioxidant action of polypodium leucotomos extract and kojic acid: reactions with reactive oxygen species Braz J Med Biol Res 2001 34 11 1487 1494 11668361 10.1590/s0100-879x2001001100018

[B36] Jayashree T Subramanyam C Oxidative stress as a prerequisite for aflatoxin production by *Aspergillus parasiticus* Free Radic Biol Med 2000 29 10 981 985 10.1016/S0891-5849(00)00398-1 11084286

[B37] Jayashree T Subramanyam C Antiaflatoxigenic activity of eugenol is due to inhibition of lipid peroxidation Lett Appl Microbiol 1999 28 3 179 183 10.1046/j.1365-2672.1999.00512.x 10196764

[B38] Kim JH Yu JJ Mahoney N Chan KL Molyneux RJ Varga J Bhatnagar D Cleveland TE Nierman WC Campbell BC Elucidation of the functional genomics of antioxidant-based inhibition of aflatoxin biosynthesis Int J Food Microbiol 2008 122 1–2 49 60 18166238 10.1016/j.ijfoodmicro.2007.11.058

[B39] Tzanidi C Proestos C Markaki P Saffron (*Crocus sativus L.*) inhibits aflatoxin B1 production by *Aspergillus parasiticus* Adv Microbiol 2012 2 3 310 316 10.4236/aim.2012.23037

[B40] Yoshinari T Akiyama T Nakamura K Kondo T Takahashi Y Muraoka Y Nonomura Y Nagasawa H Sakuda S Dioctatin A is a strong inhibitor of aflatoxin production by *Aspergillus parasiticus* Microbiology 2007 153 8 2774 2780 10.1099/mic.0.2006/005629-0 17660441

[B41] Basappa SC Sreenivasamurthy V Parpia HA Aflatoxin and kojic acid production by resting cells of *Aspergillus flavus* Link J Gen Microbiol 1970 61 1 81 86 10.1099/00221287-61-1-81 5489065

[B42] Sekiguchi J Gaucher GM Conidiogenesis and secondary metabolim in *Penicillium urticae* Appl Environ Microbiol 1977 33 1 147 158 836020 10.1128/aem.33.1.147-158.1977 PMC170614

[B43] GuzmandePena D RuizHerrera J Relationship between aflatoxin biosynthesis and sporulation in *Aspergillus parasiticus* Fungal Genet Biol 1997 21 2 198 205 10.1006/fgbi.1996.0945 9228788

[B44] Hicks JK Yu JH Keller NP Adams TH *Aspergillus* sporulation and mycotoxin production both require inactivation of the FadA G alpha protein-dependent signaling pathway EMBO J 1997 16 16 4916 4923 10.1093/emboj/16.16.4916 9305634 PMC1170127

[B45] Chang PK Hua SS Molasses supplementation promotes conidiation but suppresses aflatoxin production by small sclerotial *Aspergillus flavus* Lett Appl Microbiol 2007 44 2 131 137 10.1111/j.1472-765X.2006.02056.x 17257250

[B46] Keller NP Nesbitt C Sarr B Phillips TD Burow GB pH regulation of sterigmatocystin and aflatoxin biosynthesis in *Aspergillus spp* Phytopathology 1997 87 6 643 648 10.1094/PHYTO.1997.87.6.643 18945083

