# Supplementary figures and images for: The non-metabolizable glucose analog D-glucal inhibits aflatoxin biosynthesis and promotes kojic acid production in Aspergillus flavus
Source: BMC Microbiol. 2014 Apr 17;14:95. doi: 10.1186/1471-2180-14-95 (PMC4021404; doi:10.1186/1471-2180-14-95)

## Slide 1
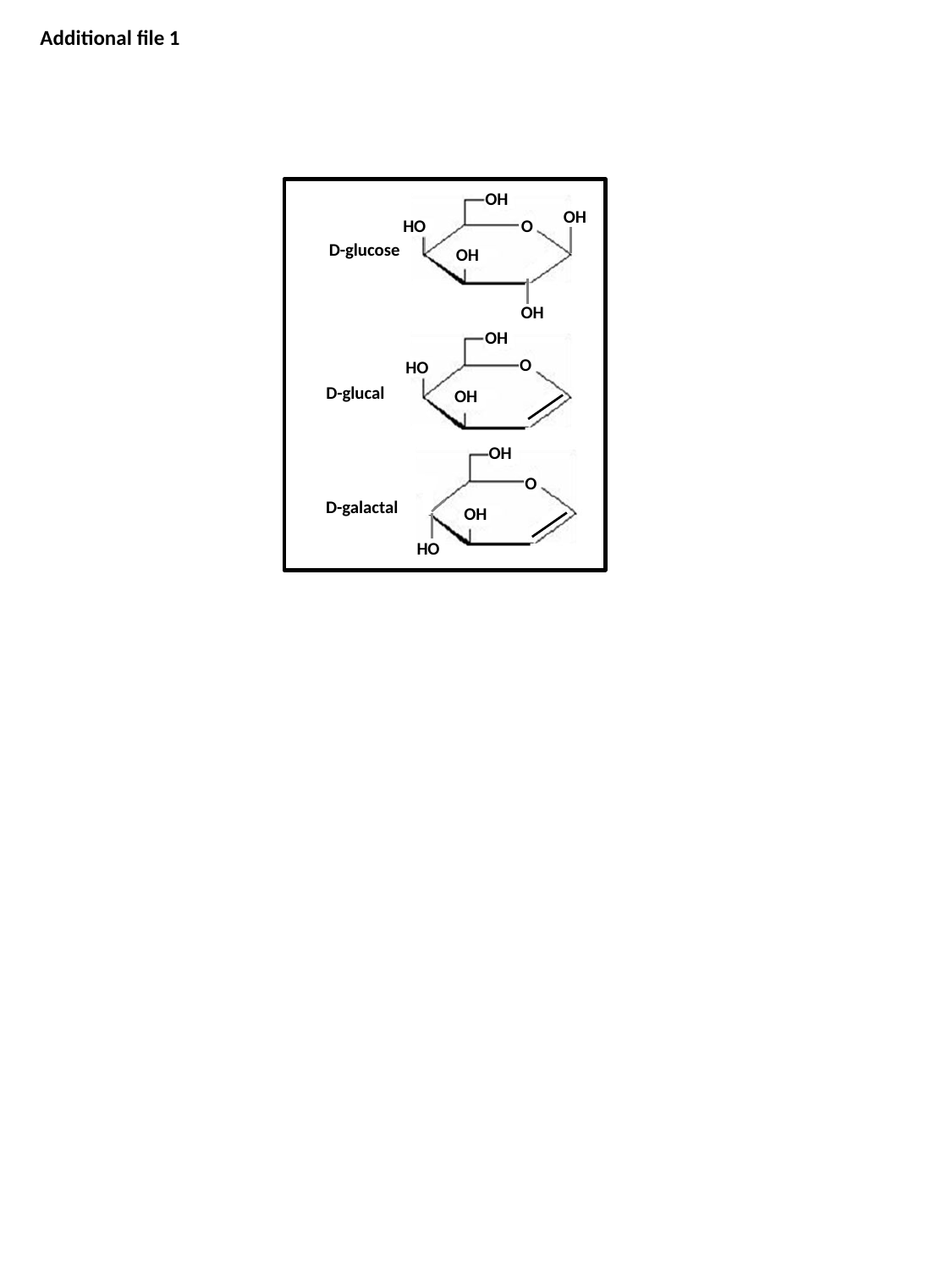

Additional file 1
OH
O
OH
HO
D-glucose
OH
OH
OH
O
HO
OH
D-glucal
OH
O
OH
HO
D-galactal

Supplement: Additional file 1 — Structures of D-glucose, D-glucal and D-galactal. [file 1471-2180-14-95-S1.pptx]
