# Supplementary material for: The non-metabolizable glucose analog D-glucal inhibits aflatoxin biosynthesis and promotes kojic acid production in Aspergillus flavus
Source: BMC Microbiol. 2014 Apr 17;14:95. doi: 10.1186/1471-2180-14-95 (PMC4021404; doi:10.1186/1471-2180-14-95)
Supplement: Additional file 2: Table S1 — Primers used for qRT-PCR. [file 1471-2180-14-95-S2.pptx]

## Slide 1
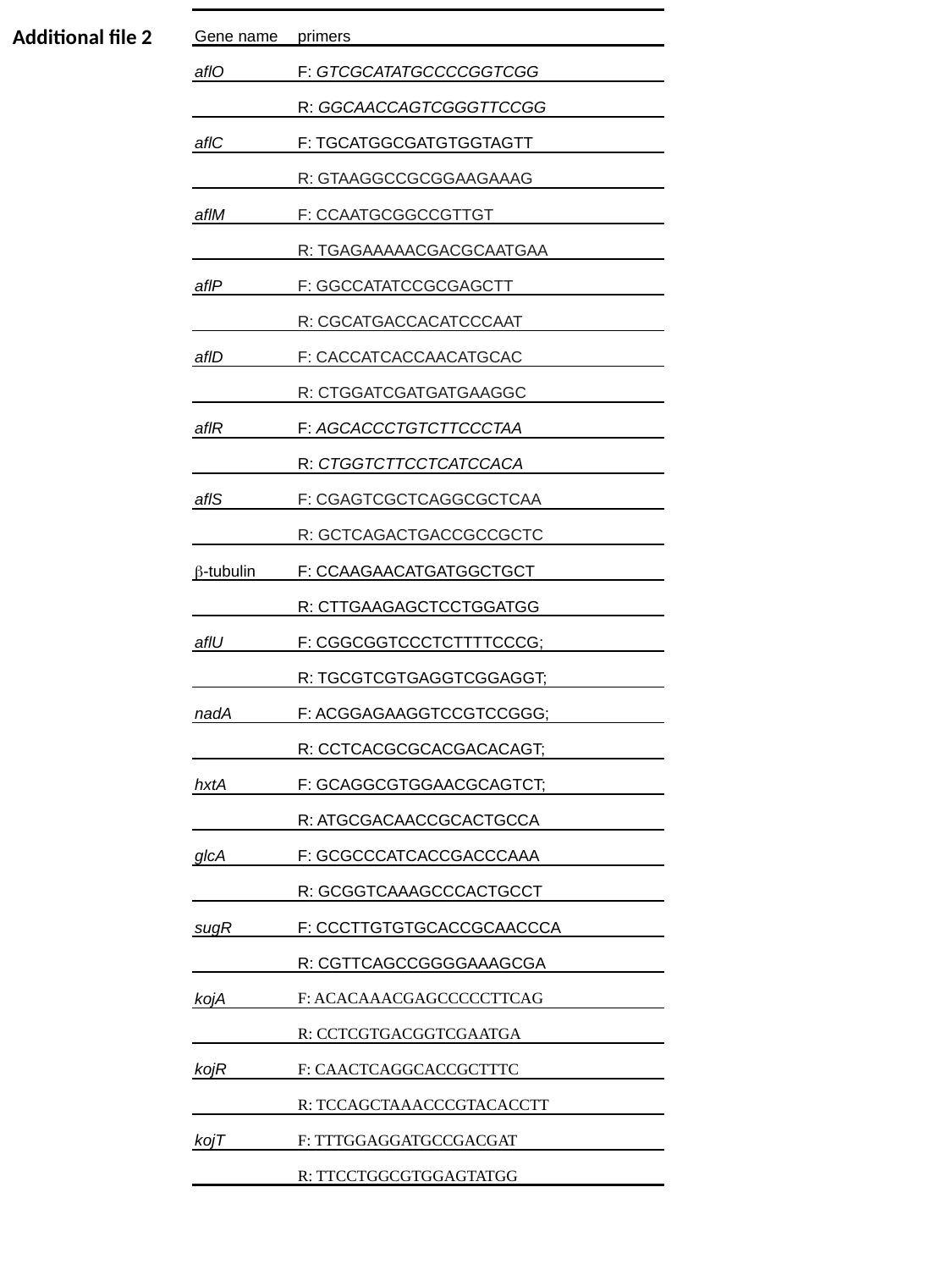

| Gene name | primers |
| --- | --- |
| aflO | F: GTCGCATATGCCCCGGTCGG |
| | R: GGCAACCAGTCGGGTTCCGG |
| aflC | F: TGCATGGCGATGTGGTAGTT |
| | R: GTAAGGCCGCGGAAGAAAG |
| aflM | F: CCAATGCGGCCGTTGT |
| | R: TGAGAAAAACGACGCAATGAA |
| aflP | F: GGCCATATCCGCGAGCTT |
| | R: CGCATGACCACATCCCAAT |
| aflD | F: CACCATCACCAACATGCAC |
| | R: CTGGATCGATGATGAAGGC |
| aflR | F: AGCACCCTGTCTTCCCTAA |
| | R: CTGGTCTTCCTCATCCACA |
| aflS | F: CGAGTCGCTCAGGCGCTCAA |
| | R: GCTCAGACTGACCGCCGCTC |
| b-tubulin | F: CCAAGAACATGATGGCTGCT |
| | R: CTTGAAGAGCTCCTGGATGG |
| aflU | F: CGGCGGTCCCTCTTTTCCCG; |
| | R: TGCGTCGTGAGGTCGGAGGT; |
| nadA | F: ACGGAGAAGGTCCGTCCGGG; |
| | R: CCTCACGCGCACGACACAGT; |
| hxtA | F: GCAGGCGTGGAACGCAGTCT; |
| | R: ATGCGACAACCGCACTGCCA |
| glcA | F: GCGCCCATCACCGACCCAAA |
| | R: GCGGTCAAAGCCCACTGCCT |
| sugR | F: CCCTTGTGTGCACCGCAACCCA |
| | R: CGTTCAGCCGGGGAAAGCGA |
| kojA | F: ACACAAACGAGCCCCCTTCAG |
| | R: CCTCGTGACGGTCGAATGA |
| kojR | F: CAACTCAGGCACCGCTTTC |
| | R: TCCAGCTAAACCCGTACACCTT |
| kojT | F: TTTGGAGGATGCCGACGAT |
| | R: TTCCTGGCGTGGAGTATGG |
Additional file 2
